# Pretransplant dyslipidaemia determines outcome in lung transplant recipients

**DOI:** 10.1186/1476-511X-12-53

**Published:** 2013-04-23

**Authors:** Urs Wenger, Silvia R Cottini, Georg Noll, Stefan Arndt, Paul A Stehberger, Stefanie Klinzing, Reto A Schuepbach, Markus Béchir

**Affiliations:** 1 Surgical Intensive Care Medicine, University Hospital of Zurich, Zurich, Switzerland; 2 Department of Cardiology, University Hospital of Zurich, Zurich, Switzerland

**Keywords:** Lung transplantation, Mortality, Lipids, Lipoproteins, Cardiovascular morbidity and cardiovascular disease

## Abstract

**Background:**

There is little knowledge about the effect of dyslipidaemia on the outcome after lung transplantation. Thus, the aim of this retrospective single centre study was to analyse the impact of the plasma lipid profile on mortality in lung transplant recipients. From January 2000 to December 2008 the charts of 172 consecutive lung transplantation recipients were analysed. At baseline and after one year lipid profiles were routinely collected. During the follow-up major cardiovascular events (MCE; beginning of dialysis, cerebrovascular insult or myocardial infarction) were recorded. The follow-up period ended December 2010.

**Findings:**

Over all total cholesterol (4.3 ± 1.6 vs. 5.4 ± 1.3 mmol/l, p < 0.0001), triglycerides (1.2 ± 0.7 vs. 2.4 ± 1.3 mmol/l, p < 0.0001), HDL (1.5 ± 0.6 vs. 1.7 ± 0.6 mmol/l, p = 0.003) and TC/HDL ratio (3.0 ± 1.0 vs. 3.6 ± 1.2, p = 0.002) increased significantly after 1 year.

During the observational period 6.9% (10 patients) suffered a major cardiac event. In univariate analysis MCE was associated with baseline TC: on average the event-group had a 33% higher baseline TC (5.6 vs. 4.2 mmol/l, OR 1.6, CI 1.1 – 2.2, p = 0.02). The total mortality in the observational period was 25% (36 patients overall). In univariate analysis mortality was associated with increased TC/HDL ratio. The non-survivors had on average a 22% higher baseline TC/HDL ratio (3.6 vs. 2.8, HR 2.8, CI 1.2 – 3.5, p = 0.001). There was no association between mortality and TC (p = 0.33), triglycerides (p = 0.34), HDL (p = 0.78) and creatinine (p = 0.73). In a multivariate model the hazard ratio was 1.5 (1.2 – 1.9, p = 0.001) per increase of 0.4 TC/HDL ratio.

**Conclusions:**

This study shows that the total cholesterol before transplantation is associated with the incidence of MCE and the cholesterol/HDL ratio with mortality in lung transplanted recipients.

## Findings

In the general population, dys- and hyperlipidemia are major risk factors for cardiovascular diseases [1]. Less is known about the importance of hyperlipidemia in lung transplant recipients. Silverborn and colleagues have shown that 5 years after lung transplantation 48% of the patients developed a new hypercholesterolemia [2]. This was recently confirmed by Nash et al. [3] and by Reed et al. [4]. The later group compared lung transplant recipients with chronic obstructive pulmonary disease (COPD) to ones with other underlying diseases. With or without COPD after lung transplantation the triglyceride levels were significantly increased relative to pre-transplantation. Interestingly the high-density lipoproteins (HDL) values and the ratios of low-density lipoproteins (LDL) versus HDL and total cholesterol (TC) versus HDL increased significantly only in the COPD group. Whether these changes have an impact on the further disease course remains unclear. In a cohort of 230 lung transplant recipients, Stephany et al. demonstrated a strong association between early post-transplant LDL values and renal function – the higher the former the worser the latter [5]. But the height of serum LDL did not significantly correlate with mortality. In the aforementioned study of Nash and colleagues the estimated 10-years cardiovascular risk (Framingham risk score) was low (< 10% in all but one included patients – 87 out of 88) despite the changes in lipid profile. The aim of our retrospective study was to asses the impact of baseline serum lipid levels on outcome in lung transplant recipients.

Following approval by the local Ethics Committee, which waived the need for written informed consent for this post hoc data analysis, a systematic chart review on 172 consecutive adult lung recipients transplanted from January 2000 to December 2008 was performed. Clinical and demographic data and lipid profiles (TC, triglycerides, HDL, TC/HDL ratio, all values were fasting plasma lipids) were collected at listing time (baseline) and at around one year post transplantation, corresponding to routine outpatient visits. None of the patients was on statin medication. Follow-up data included occurrence of major cardiovascular event (MCE; defined as need for dialysis, cerebrovascular insult or myocardial infarction) and all-cause mortality. The observational period ended December 2010.

The immunosuppression protocol used at our institution included a calcineurin inhibitor (CNI) with an antimetabolite (azathioprine or mycophenolate mofetil) and steroids.

Baseline values were compared with the 1-year follow up values with Wilcoxon rank test. Univariate and step down multivariate logistic regression was done for major cardiovascular events and a cox proportional model for survival (Covariates were age, underlying disease, gender; keratinize before TPL and the lipid profiles). All calculations were done with Stat view 4.5 (abacus concepts, Berkeley, CA, USA). Statistical significance was accepted with p < 0.05 (two sided tests).

**Figure 1:**
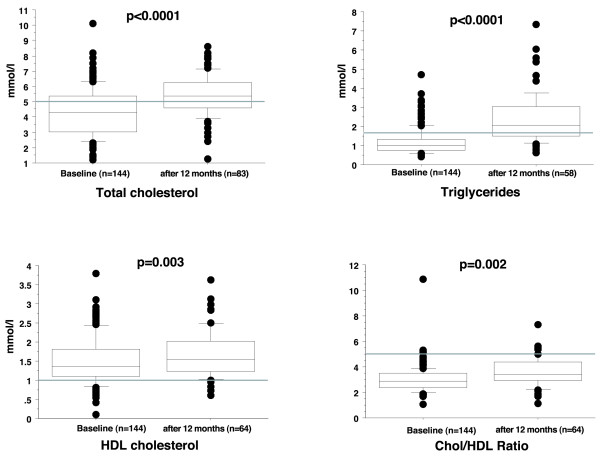
**As shown in these box-plots (the box outlining 25**^**th **^**and 75**^**th **^**percentile, line showing median and whiskers depicting 90**^**th **^**percentile) comparing the baseline values with the values after one year of the whole study population TC, triglycerides, HDL-cholesterol and TC/HDL ratio increased significantly after 1 year.** The grey line indicates the border of normal values, beneath the line are normal values in TC, triglycerides and TC/HDL ratio, above the line for HDL. HDL indicates high-density lipoprotein and TC total cholesterol.

From the 172 consecutive patients twenty-eight had to be omitted because of incomplete baseline data. Finally 144 lung transplant recipients with a minimal follow up of two years were analyzed. Baseline characteristics of the whole study population and each of the subgroups (survivors vs. non-survivors, with MCE vs. without MCE) are shown on Table 1. Over all TC (4.3 ± 1.6 vs. 5.4 ± 1.3 mmol/l, p < 0.0001), triglycerides (1.2 ± 0.7 vs. 2.4 ± 1.3 mmol/l, p < 0.0001), HDL (1.5 ± 0.6 vs. 1.7 ± 0.6 mmol/l, p = 0.003) and TC/HDL ratio (3.0 ± 1.0 vs. 3.6 ± 1.2, p = 0.002) increased significantly after 1 year – for details see Figure 1.

**Table 1:** Baseline characteristics

	**All**	**Survivors**	**Nonsurvivors**	**P**	**Without MCE**	**with MCE**	**P**
	**(n = 144)**	**(n = 108)**	**(n = 36)**		**(n = 134)**	**(n = 10)**	
Men/Women	84 (58.3%)/60 (41.7%)	66/42	25/11	0.56	81/53	7/3	0.78
Age → (yrs.)	44.8 ± 16.4	44.7 ± 16.5	49.9 ± 14.3	0.10	45.6 ± 16.1	50.9 ± 13.8	0.40
Weight (kg)	60.6 ± 16.6	58.9 ± 15.1	61.0 ± 14.2	0.81	59.6 ± 15.1	60.5 ± 13.9	0.69
Height (m)	1.67 ± 0.09	1.68 ± 0.09	1.71 ± 0.10	0.69	1.68 ± 0.09	1.71 ± 0.05	0.57
BMI (kg/m^2^)	21.6 ± 5.0	22.1 ± 4.2	23.5 ± 4.9	0.59	22.0 ± 4.9	22.9 ± 5.1	0.67
Creatinine (μmol/l)	75 ± 19	75 ± 18	76 ± 19	0.73	74 ± 19	87 ± 15	0.03
Urea (mmol/l)	4.4 ± 1.9	4.7 ± 1.8	4.9 ± 2.1	0.59	4.7 ± 1.8	5.4 ± 2.1	0.27
TC (mmol/l)	4.3 ± 1.6	4.2 ± 1.6	4.5 ± 1.5	0.33	4.2 ± 1.5	5.6 ± 2.0	0.007
Triglycerides (mmol/l)	1.2 ± 0.7	1.2 ± 0.7	1.3 ± 0.8	0.34	1.2 ± 0.7	1.0 ± 0.4	0.28
HDL (mmol/l)	1.5 ± 0.6	1.5 ± 0.6	1.5 ± 0.7	0.78	1.5 ± 0.6	1.9 ± 0.9	0.01
TC/HDL Ratio	3.0 ± 1.0	2.8 ± 0.7	3.6 ± 1.7	0.001	3.0 ± 1.1	2.2 ± 0.6	0.67
Underlying diseases							
CF	47 (32.6%)	40	7		46	1	
COPD	42 (29.2%)	30	12		37	5	
IPF	30 (20.8%)	20	10	0.19	28	2	0.67
PAH	9 (6.3%)	3	6		9	0	
Miscellaneous	16 (11.1%)	12	4		14	2	

Data expressed as mean ± SD. Underlying diseases expressed as no. of patients and percentage of total no. of patients. BMI indicates body mass index, COPD chronic obstructive pulmonary disease, CF cystic fibrosis, HDL high-density lipoprotein, IPF idiopathic pulmonary fibrosis, PAH pulmonary arterial hypertension and TC total cholesterol. Paired T-test or chi-squared Test.

During the observational period 6.9% (10 patients) suffered a MCE (Table 1). In univariate analysis MCE was associated with baseline TC: on average the event-group had a 33% higher baseline TC (5.6 vs. 4.2 mmol/l, OR 1.6, CI 1.1 – 2.2, p = 0.02). Interestingly the HDL was significantly higher (1.5 vs. 1.9 mmol/l, p = 0.01) in the patients that suffered a MCE. Furthermore, there was no association with the TC/HDL ratio (p = 0.67) and triglycerides (p = 0.28). There was also an association between MCE and creatinine baseline level (74 vs. 87 μmol/l, p = 0.03): Per increase of 3 mol/l baseline creatinine there was and odds ratio of 1.03 (CI 1.01 – 1.07, p = 0.04). In a multivariate model the odds ratio per increased 1.0 mmol/l total cholesterol was 2.5 (1.2 – 6.1, p = 0.01).

The total mortality in the observational period was 25% (36 patients overall). Cause of death were primary graft dysfunction (n = 8), pneumonia (n = 3), sepsis (n = 12), carcinoma (n = 1), chronic rejection (n = 4), MCE (n = 4) and misc (n = 4). In univariate analysis mortality was associated with increased TC/HDL ratio. The non-survivors had on average a 22% higher baseline TC/HDL ratio (3.6 vs. 2.8, HR 2.8, CI 1.2 – 3.5, p = 0.001). There was no association between mortality and TC (p = 0.33), triglycerides (p = 0.34), HDL (p = 0.78) and creatinine (p = 0.73). In a multivariate model the hazard ratio was 1.5 (1.2 – 1.9, p = 0.001) per increase of 0.4 TC/HDL ratio.

Our study suggests that mortality and the incidence of MCE is associated with increased pretransplant baseline lipid levels in lung transplant recipients, more precisely TC/HDL rate was associated with a higher mortality and increased TC was associated with the rate of MCE. Importantly the baseline ratio of TC/HDL of survivors and non-survivors were in the normal range even though significantly different from each other – whereas the baseline TC was in the ones that suffered from a MCE above norm. While not statistically significant, it is noteworthy that MCE victims had a notably reduced TC/HDL ratio compared to non-victims.

The underlying patho-mechanisms of hyperlipidemia for mortality and cardiovascular events in lung transplant recipients are probably the same as described in other patient groups influence on cholesterol transport, promotion of inflammation, endothelial effects, production of mediators and thrombogenicity [4].

Interpretation of the higher HDL values after one year and in the patients suffering from a MCE is difficult. Higher HDL values after renal transplantation were also seen in the study of Tse et al. [6]. The exact mechanism of rising HDL after transplantation is so far to our knowledge unclear.

Certainly the value of this evidence is limited due to its retrospective character, but as long as we lack prospective study data these findings might serve as rationale for further prospective research as well as an argument starting lipid lowering medication even before lung transplantation. Beginning a cholesterol lowering therapy after transplantation as have Johnson et al. demonstrated resulted in significantly better survival, fewer episodes of and less severe graft rejection [7]. They also showed that statin medication was associated with decrements in long-term maintenance calcineurin inhibitor dose as well as lower steroid dose supporting the growing evidence of immune modulatory effects of statins. Recently Li and coworkers have shown in a single center retrospective study via propensity score matching that pravastatin was associated with the preservation of vital capacity and hinders the development of bronchiolitis obliterans syndrome [8]. Furthermore, this data demonstrates a prolonged survival with the use of pravastatin.

Taken together the actual body of evidence even in absence of a prospective randomized controlled trial might support the use of statins in lung transplant recipients – even before transplantation.

## Competing interests

The authors declare that they have no competing interests.

## Authors’ contributions

UW, PAS, GN and SRC: Data Analysis, Interpretation and drafting the article. SA and RAS: Statistics and critical review. MB: Conception, interpretation, drafting the article and critical review SK made the revision of the paper. The submitted version was approved by all authors.
